# Supplementary material for: Phase separation and zinc-induced transition modulate synaptic distribution and association of autism-linked CTTNBP2 and SHANK3
Source: Nat Commun. 2022 May 13;13:2664. doi: 10.1038/s41467-022-30353-0 (PMC9106668; doi:10.1038/s41467-022-30353-0)
Supplement: Supplementary file 1 — Supplementary Information [file 41467_2022_30353_MOESM1_ESM.pdf]

Supplementary Information for

Phase separation and zinc-induced transition modulates synaptic distribution and association of autism-linked CTTNBP2 and SHANK3

Pu-Yun Shih, Yu-Lun Fang, Sahana Shankar, Sue-Ping Lee, Hsiao-Tang Hu, Hsin Chen, Ting-Fang Wang, Kuo-Chiang Hsia* and Yi-Ping Hsueh*

* Correspondence and requests for materials should be addressed to Yi-Ping Hsueh (yph@gate.sinioca.edu.tw) and Kuo-Chiang Hsia (khsia@gate.sinica.edu.tw)

The PDF file includes Supplementary figures, legends and references:

- **Supplementary Figure S1.** *In silico* structural prediction of wild-type (WT) and ASD-linked CTTNBP2 mutant proteins (related to **Figure 1**).
- **Supplementary Figure S2.** CTTNBP2 forms condensates in different cells (related to **Figure 1**, **Video S1** and **S2**).
- **Supplementary Figure S3.** Purified full-length CTTNBP2 and its response to zinc in solution (related to **Figures 1, 2**).
- **Supplementary Figure S4.** ASD-linked mutations differentially influence the degree of disorder and condensate formation ability of CTTNBP2 (related to **Figure 3**).
- **Supplementary Figure S5.** FRAP analyses and feature summary of ASD-linked CTTNBP2 mutants (related to **Figures 3, 4, 5**).
- **Supplementary Figure 6.** CTTNBP2 and SHANK3 form condensates in COS1 cells (related to **Figure 8**).
- **Supplementary Figure S7.** Zinc and 1,6-HD do not alter CTTNBP2 expression levels in cultured cortical neurons at DIV 18 (related to **Figure 8**).

Supplemental figures and figure legends

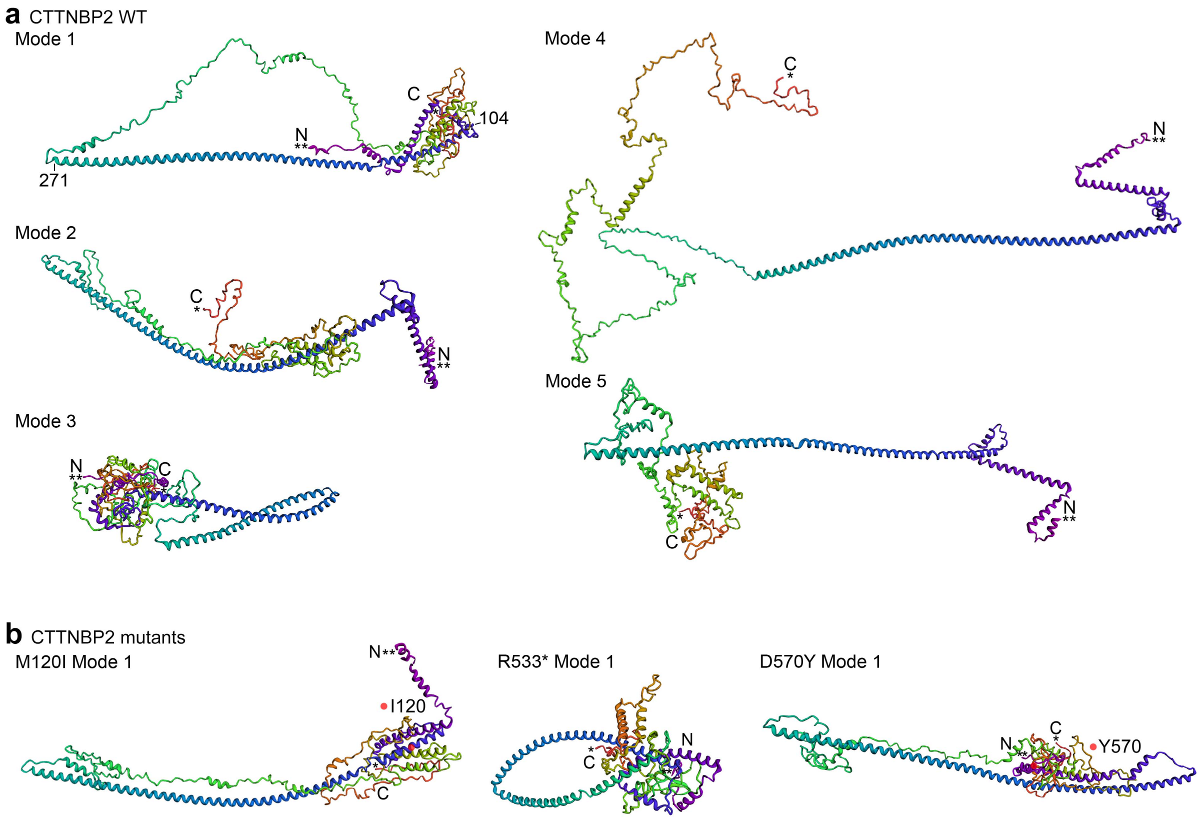

Supplementary figure 1. ***In silico*** **structural prediction of wild-type (WT) and ASD-linked CTTNBP2 mutant proteins (related to Figure 1)**

(**a**) Robetta-based prediction (https://robetta.bakerlab.org/) of WT CTTNBP2 protein structure. Five predicted modes are shown. (**b**) Robetta-based predicted structures of the ASD-linked CTTNBP2 mutant proteins, M120I, R533* and D570Y. Only Mode 1 is shown for each mutant.

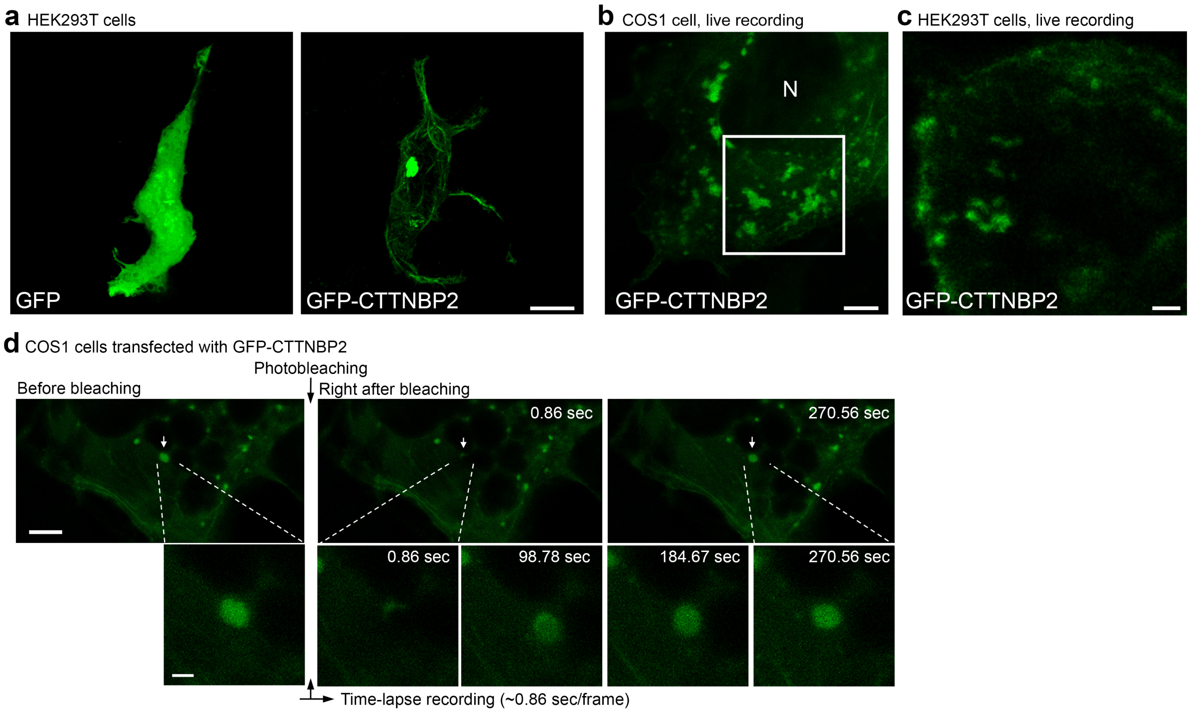

Supplementary figure 2. CTTNBP2 forms condensates in different cells (related to Figure 1, Video S1 and **S2).**

(**a**) GFP-CTTNBP2 forms condensates in HEK293T cells. GFP was used as a control. (**b-c**) Whole-cell live-image of GFP-CTTNBP2 in COS1 (**b**) and HEK293T (**c**) cells. These images r epresent the starting point of Videos S1 and S2, respectively. (**d**) Molecular exchange between the cytosol and CTTNBP2 condensate. The arrows point to the isolated CTTNBP2 condensate that was photobleached. Live recording was performed every 0.86 sec/frame. Scale bar: (**a**) 10 μm; (**b**) 5 μm; (**c**) 2 μm; (**d**) upper 10 μm, lower 2 μm.

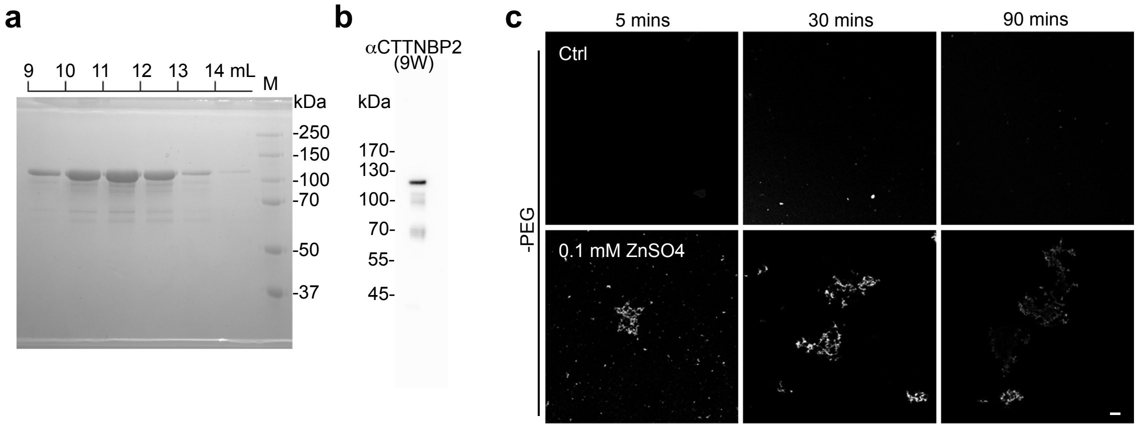

Supplementary figure 3. Purified full-length CTTNBP2 and its response to zinc in solution (related to Figures 1 and 2).

(**a**) Peak fractions of dual-tagged full-length His-GFP-CTTNBP2 in a superdex 200 column, as visualized by Coomassie blue staining. The molecular weight marker and elution volume of each fraction are indicated. (**b**) Immunoblotting using anti-CTTNBP2 antibody (9W) to validate purified GFP-CTTNBP2. (**c**) Zinc-induced CTTNBP2 aggregate formation is independent of PEG. Scale 2 μm.

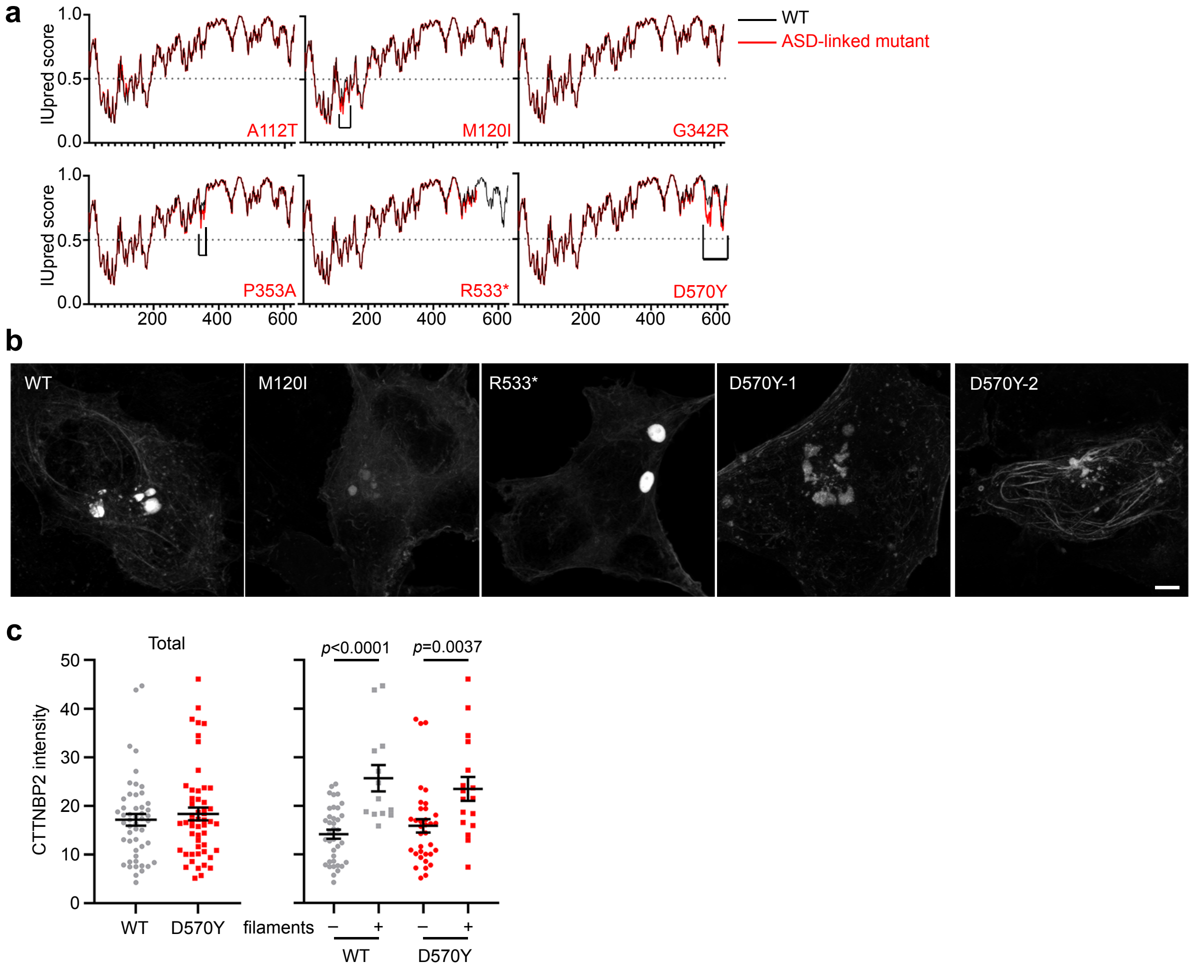

Supplementary figure 4. ASD-linked mutations differentially influence the degree of disorder and condensate formation ability of CTTNBP2 (related to Figure 3)

(**a**) Degrees of disorder for ASD-linked CTTNBP2 mutant proteins, as predicted using IUpred. For each plot, the WT (black line) and mutant (red line) profiles largely overlap, but some regions exhibiting differences are indicated. (**b**) GFP-tagged WT and ASD-linked CTTNBP2 mutant proteins form condensates in COS1 cells. Some condensates are rounded and some are irregular. As the D570Y mutant displays a higher affinity for microtubules [1], the microtubule-like distribution pattern was much clearer for D570Y mutant-expressing cells, even though the D570Y mutant can still form condensates. In the panel, D570Y-1 represents an example showing multiple large droplet-like condensates and D570Y-2 is an example showing a microtubule-associated CTTNBP2 distribution. Note that WT and the other mutant proteins can also associate with microtubules, though the respective signals were weaker. (**c**) The micro tubule-associated distribution pattern of CTTNBP2 is linked to a higher expression level of CT TNBP2. We further quantified the intensity of CTTNBP2 immunoreactivity. There was no difference in total immunoreactivities between WT and the D570Y mutant. However, we observed that cells exhibiting the microtubule-associated distribution pattern tended to have a higher total intensity of CTTNBP2 compared to cells without that pattern, no matter whether

WT or D570Y mutant protein was being considered. Mann Whitney test, two-sided. **, $p < 0.01$; ***, $p < 0.001$. Scale bar: 5 μm.

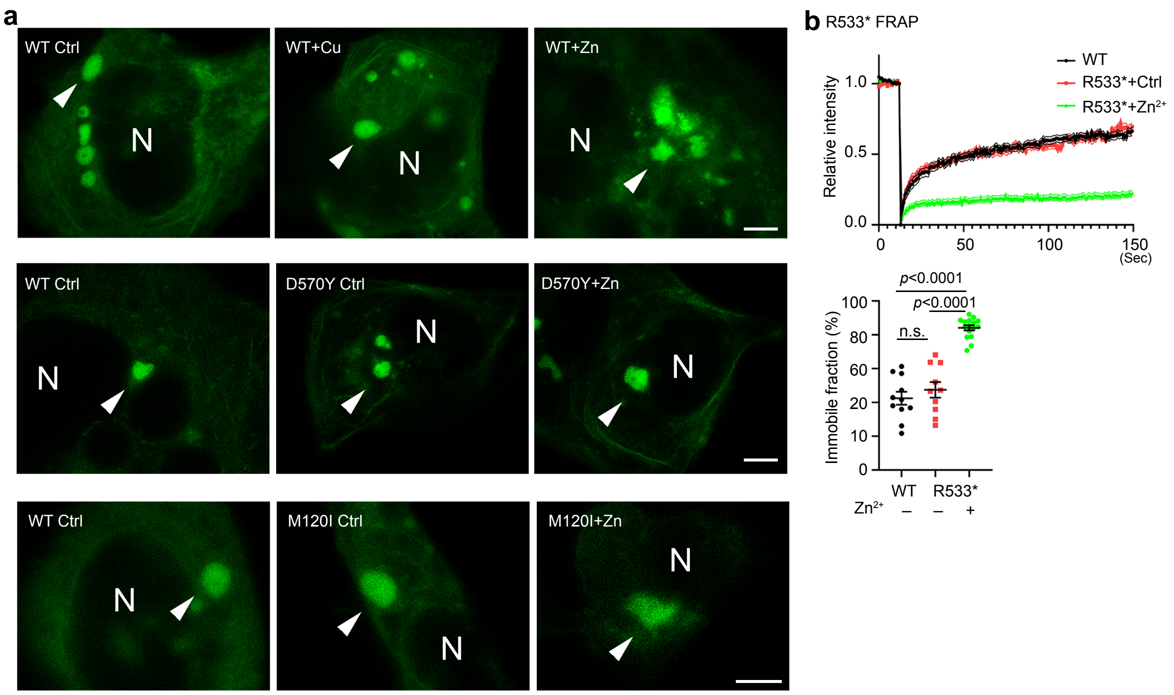

c

		D570Y	R533*	M120I
	Protein interaction	Microtubule binding↑	No cortactin binding	Cortactin binding ↓<br>N-C terminal interaction ↓
	Dendritic spine formation *in vivo*	Density ↓	Density ↓<br>Width ↓	Density ↓<br>Width and length ↓
In solution	Size of condensates	Bigger in crude lysates<br>Smaller in exp. using purified proteins	Smaller	Smaller
In solution	Zinc response	Yes	Yes	Yes
In COS1	Mobility	Slower	The same	Much faster
In COS1	Immobile fraction	The same	The same	Decreased
In COS1	Zinc effect	Immobilized	Immobilized	Immobilized

Supplementary figure 5. FRAP analyses and feature summary of ASD-linked CTTNBP2 mutant proteins (related to Figures 3, 4 and 5).

(**a**) Cell images of FRAP analysis described in Figure 3. The images of the starting point for each time-lapse recording are shown. (**b**) FRAP of the GFP-tagged R533* mutant protein in COS1 cells. Upper, relative intensity throughout the experiment. Lower, immobile fractions of the WT and R533* mutant proteins are comparable. However, zinc significantly increases the immobile fraction of the R533* mutant. Data are presented as mean values +/- SEM and the individual data point. N=14 (WT), 10 (R533*) and 16 (R533*+Zn) cells collected from more than three independent experiments. One-way ANVOA with Bonferroni's multiple comparisons test; ***, $p < 0.001$. (**c**) Summary of the diverse features of the purified ASD-linked CTTNBP2 mutant proteins, i.e. D570Y, R533* and M120I. Molecular and morphological features from current and previous studies [1, 2] are listed. The features are described relative to WT CTTNBP2.

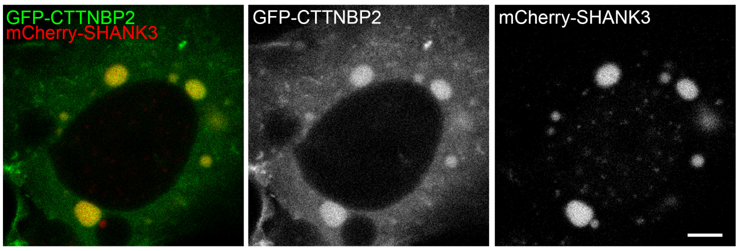

Supplemetary figure 6. CTTNBP2 and SHANK3 form condensates in COS1 cells (related to Figure 8)

GFP-CTTNBP2 and mCherry-SHANK3 were transfected into COS1 cells and visualized by confocal microscopy. Scale 5 µm.

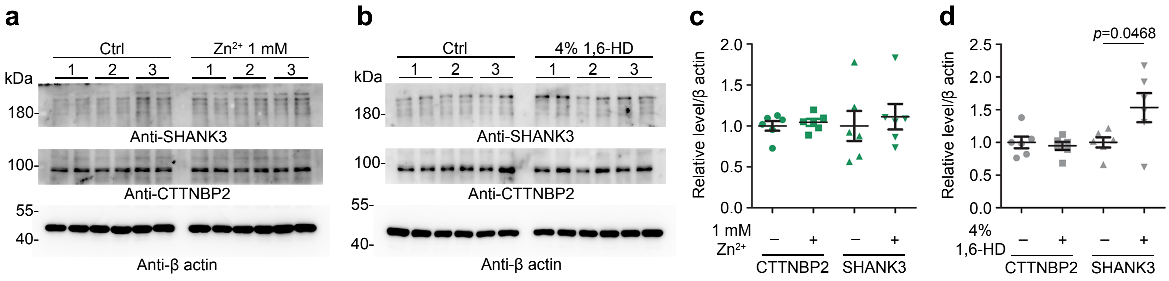

Supplemetary figure S7. Zinc and 1,6-HD do not alter CTTNBP2 expression levels in cultured cortical neurons at DIV 18 (related to Figure 8).

(**a**) Expression levels of CTTNBP2 and SHANK3 proteins in the absence or presence of 1 mM $ZnSO_4$. β-actin was used as an internal control. (**b**) Expression levels of CTTNBP2 and SHANK3 proteins upon treatment with 4% 1,6-HD and 5 µg/ml digitonin. Digitonin alone was used as a control (Ctrl). β-actin acted as an internal control. Although the protein levels of CTTNBP2 were not changed, the protein levels of SHANK3 were increased in the presence of 1,6-HD for unknown reasons. (**c**) Quantification of (**a**). (**d**) Quantification of (**b**). (**c**) and (**d**) N=6 independent preparations. Data are presented as mean values +/- SEM and the results of individual data points. Two-tailed unpaired *t* test. *, $p < 0.05$.

References:

1. Shih, P.Y. *et al.* Autism-linked mutations of CTTNBP2 reduce social interaction and impair dendritic spine formation via diverse mechanisms. *Acta neuropathologica communications* **8**, 185 (2020).
2. Shih, P.Y. *et al.* CTTNBP2 Controls Synaptic Expression of Zinc-Related Autism-Associated Proteins and Regulates Synapse Formation and Autism-like Behaviors. *Cell reports* **31**, 107700 (2020).
