# Supplementary material for: Phase separation and zinc-induced transition modulate synaptic distribution and association of autism-linked CTTNBP2 and SHANK3
Source: Nat Commun. 2022 May 13;13:2664. doi: 10.1038/s41467-022-30353-0 (PMC9106668; doi:10.1038/s41467-022-30353-0)
Supplement: Supplementary file 3 — Description of additional Supplementary File [file 41467_2022_30353_MOESM3_ESM.pdf]

Descriptions of Additional Supplementary Files

Supplementary Movie 1. Mobile condensates of GFP-tagged CTTNBP2 in a COS1 cell (related to Figures 1c, S2b).

Supplementary Movie 2. Mobile condensates of GFP-tagged CTTNBP2 in a HEK293T cell (related to Figure S2c).

Supplementary Movie 3-5. Three examples of the fusion behavior of GFP-tagged CTTNBP2 condensates in solution with 3% PEG (related to Figure 1j).

Supplementary Movie 6. GFP-tagged CTTNBP2 aggregates in the presence of 3% PEG and 0.1 mM zinc (related to Figure 2h).

Supplementary Movie 7. GFP-tagged CTTNBP2 aggregates in the presence of 3% PEG only (related to Figure 2h).

Supplementary Movie 8. FRAP of GFP-tagged WT CTTNBP2 without further treatment (related to Figure 3a).

Supplementary Movie 9. FRAP of GFP-tagged WT CTTNBP2 in the presence of 1 mM zinc (related to Figure 3a).

Supplementary Movie 10. FRAP of GFP-tagged WT CTTNBP2 in the presence of 1 mM copper (related to Figure 3a).

Supplementary Movie 11. FRAP of GFP-tagged WT CTTNBP2 (related to Figure 3b).

Supplementary Movie 12. FRAP of GFP-tagged D570Y CTTNBP2 (related to Figure 3b).

Supplementary Movie 13. FRAP of GFP-tagged D570Y CTTNBP2 in the presence of 1 mM zinc (related to Figure 3b).

Supplementary Movie 14. FRAP of GFP-tagged WT CTTNBP2 (related to Figure 3c).

Supplementary Movie 15. FRAP of GFP-tagged M120I CTTNBP2 (related to Figure 3c).

Supplementary Movie 16. FRAP of GFP-tagged M120I CTTNBP2 in the presence of 1 mM zinc (related to Figure 3c).

Supplementary Movie 17. Purified GFP-CTTNBP2 and mCherry-SHANK3 co-condense in solution (related to Figure 8a).

Supplementary Movie 18. Dual immunostaining of CTTNBP2 (red) and SHANK3 (green) in mature hippocampal culture. In the Imarisgenerated 3D model, the CTTNBP2 aggregates have been rendered semi-transparent to reveal how they intermingle with SHANK3 (related to Figure 8d).
