# Supplementary material for: Phase separation and zinc-induced transition modulate synaptic distribution and association of autism-linked CTTNBP2 and SHANK3
Source: Nat Commun. 2022 May 13;13:2664. doi: 10.1038/s41467-022-30353-0 (PMC9106668; doi:10.1038/s41467-022-30353-0)
Supplement: Supplementary file 22 — Reporting Summary [file 41467_2022_30353_MOESM22_ESM.pdf]

nature portfolio

Corresponding author(s): Yi-Ping Hsueh

Last updated by author(s): Mar 29, 2022

Reporting Summary

Nature Portfolio wishes to improve the reproducibility of the work that we publish. This form provides structure for consistency and transparency in reporting. For further information on Nature Portfolio policies, see our Editorial Policies and the Editorial Policy Checklist.

Statistics

For all statistical analyses, confirm that the following items are present in the figure legend, table legend, main text, or Methods section.

n/a	Confirmed	
☐	☒	The exact sample size (n) for each experimental group/condition, given as a discrete number and unit of measurement
☐	☒	A statement on whether measurements were taken from distinct samples or whether the same sample was measured repeatedly
☐	☒	The statistical test(s) used AND whether they are one- or two-sided<br>*Only common tests should be described solely by name; describe more complex techniques in the Methods section.*
☒	☐	A description of all covariates tested
☐	☒	A description of any assumptions or corrections, such as tests of normality and adjustment for multiple comparisons
☐	☒	A full description of the statistical parameters including central tendency (e.g. means) or other basic estimates (e.g. regression coefficient) AND variation (e.g. standard deviation) or associated estimates of uncertainty (e.g. confidence intervals)
☐	☒	For null hypothesis testing, the test statistic (e.g. F, t, r) with confidence intervals, effect sizes, degrees of freedom and P value noted<br>*Give P values as exact values whenever suitable.*
☒	☐	For Bayesian analysis, information on the choice of priors and Markov chain Monte Carlo settings
☒	☐	For hierarchical and complex designs, identification of the appropriate level for tests and full reporting of outcomes
☒	☐	Estimates of effect sizes (e.g. Cohen's d, Pearson's r), indicating how they were calculated

Our web collection on statistics for biologists contains articles on many of the points above.

Software and code

Policy information about availability of computer code

Data collection	Zen black and Zen blue were employed to collect data in a unbiased manner.
Data analysis	ImageJ v1.52a and Microsoft Excel was used to analyze and quantify images. Photoshop CS3 and 2020 and Adobe Illustrator 2020 were used to process and assemble data. Statistical analysis was performed with Prism v5.03, 8.3 or 9.3.1 (Graphpad software).

For manuscripts utilizing custom algorithms or software that are central to the research but not yet described in published literature, software must be made available to editors and reviewers. We strongly encourage code deposition in a community repository (e.g. GitHub). See the Nature Portfolio guidelines for submitting code & software for further information.

Data

Policy information about availability of data

All manuscripts must include a data availability statement. This statement should provide the following information, where applicable:

- Accession codes, unique identifiers, or web links for publicly available datasets
- A description of any restrictions on data availability
- For clinical datasets or third party data, please ensure that the statement adheres to our policy

Yes, all raw data have been summarized in Supplementary Table S2.

Field-specific reporting

Please select the one below that is the best fit for your research. If you are not sure, read the appropriate sections before making your selection.

- [x] Life sciences
- [ ] Behavioural & social sciences
- [ ] Ecological, evolutionary & environmental sciences

For a reference copy of the document with all sections, see nature.com/documents/nr-reporting-summary-flat.pdf

Life sciences study design

All studies must disclose on these points even when the disclosure is negative.

Sample size	We did not statistically determine the sample size but the sample sizes we collected are comparable to published paper for neuronal morphometry and behavioral analysis. For the size of condensates, the sample size are beyond the power of statistical analysis.
Data exclusions	The outliners were identified by method "ROUT" in PRISM 8 and excluded.
Replication	For image analysis, primary culture from one litter of pregnant mice was considered as one biological replication. For COS1 cell or HEK293T cell experiment, each transfection considered as a biological replication. All experiments could be successfully repeated in at least two, usually more than three times of independent experiments.
Randomization	For behavioral analysis, all littermates including WT and mutant mice were used in the same social behavior test. Randomization was not applicable. For cultured neuron, different wells of neurons were randomly allocated into different treatment in one experiment. For biochemical assays such as ITC or droplet assay, radomization is not suitable.
Blinding	Blinded test were employed for imaging analyses (including morphology or protein distribution) and behavioral test. The investigators were blinded to group allocation during data analysis

Reporting for specific materials, systems and methods

We require information from authors about some types of materials, experimental systems and methods used in many studies. Here, indicate whether each material, system or method listed is relevant to your study. If you are not sure if a list item applies to your research, read the appropriate section before selecting a response.

Materials & experimental systems

n/a	Involved in the study	
[ ]	[x]	Antibodies
[ ]	[x]	Eukaryotic cell lines
[x]	[ ]	Palaeontology and archaeology
[ ]	[x]	Animals and other organisms
[x]	[ ]	Human research participants
[x]	[ ]	Clinical data
[x]	[ ]	Dual use research of concern

Methods

n/a	Involved in the study	
[x]	[ ]	ChIP-seq
[x]	[ ]	Flow cytometry
[x]	[ ]	MRI-based neuroimaging

Antibodies

Antibodies used	CTTNBP2 antiserum 9W (1/500) and purified antibody A5 (0.5 µg/ml) 32, 35; HA tag (Cell Signaling, C29F4, 0.5 µg/ml; Roche, 3F10, 0.5 µg/ml); GFP (Abcam, ab13970, 0.5 µg/ml; Invitrogen, A6455, 1/1000); β-actin (Sigma-Aldrich, AC-74, 0.5 µg/ml); SHANK3 (Synaptic System, 162 304, 1/500); horseradish peroxidase-conjugated secondary antibodies (GE healthcare, NA931, anti-mouse IgG, and NA934, anti-rabbit IgG; Sigma-Aldrich, A7289, anti-guinea pig IgG; all 1/5000); Alexa Fluor-conjugated secondary antibodies (Invitrogen, A-11039, anti-chicken IgY, 488; A-21206, anti-rabbit IgG, 488; A-11073, anti-guinea pig IgG, 488; A-21424, anti-mouse IgG, 555; A-21429, anti-mouse IgG, 555; A-21209, anti-rat IgG, 594; all 1/500).
Validation	Anti-CTTNBP2 9W and A7, rat and mouse, immunoblot and immunostaining, PMID: 32492416<br>Anti-β-actin, Sigma-Aldrich, AC-74, mouse, immunoblot, https://www.labome.com/review/gene/human/beta-actin-antibody.html.<br>Anti-HA tag, Cell Signaling, C29F4, immunoblot and immunostaining, PMID: 2455217, https://www.cellsignal.com/products/primary-antibodies/ha-tag-c29f4-rabbit-mab/3724<br>Anti-HA tag, Roche, 3F10, immunoblot and immunostaining, PMID:29950568, https://www.sigmaaldrich.com/TW/en/product/roche/roahaha?gclid=EAIaIQobChMI3OvNh53n9gIVGVRgCh0ZSgR8EAAYASAAEgIUOfD_BwE<br>Anti-GFP, Abcam, ab13970, immunoblot and immunostaning, PMID: 34326333, https://www.abcam.com/gfp-antibody-ab13970.html<br>Anti-GFP, invitrogen, A6455, immunoblot and immunostaining, PMID: 32938929, https://www.thermofisher.com/antibody/product/GFP-Antibody-Polyclonal/A-6455<br>Anti-Shank3, Synaptic System, 162 304, rat and mouse, immunoblot and immunostaining, PMID: 33199684 and 33115499, , https://sysy.com/product/162304

Eukaryotic cell lines

Policy information about cell lines

Cell line source(s)	HEK293T, COS1 cell, High-five insect cells (ThermoFisher, B85502)
Authentication	Cell lines used in this study were not authenticated.
Mycoplasma contamination	Mycoplasma contamination was not tested in this study.
Commonly misidentified lines (See ICLAC register)	Commonly misidentified lines were not used in this study.

Animals and other organisms

Policy information about studies involving animals; ARRIVE guidelines recommended for reporting animal research

Laboratory animals	C57BL/6 were used for culture neuron and behavioral test. All the mice for behavioral test were male and older than 8 week. (See Methods). Mixed-sex embryos at embryonic day 17-18 were used for preparation of cultured neurons<br>All animal experiments were performed with the approval of the Academia Sinica Institutional Animal Care and Utilization Committee (Protocol # 12-10-414 and 11-12-294), and in strict accordance with its guidelines and those of the Council of Agriculture Guidebook for the Care and Use of Laboratory Animals, Taiwan. Animals were housed and bred in the animal facility of the Institute of Molecular Biology, Academia Sinica, under controlled humidity (45-55%) and temperature (19-22°C) and a 12 h light/dark cycle (light off at 20:00). Animals accessed water and food (#5K54, LabDiet) ad libitum. This has been also described in the manuscript.
Wild animals	No wild animal was used in the study.
Field-collected samples	No field-collected sample was used in the study.
Ethics oversight	All animal experiments were performed with the approval of the Academia Sinica Institutional Animal Care and Utilization Committee (Protocol # 12-10-414 and 11-12-294), and in strict accordance with its guidelines and those of the Council of Agriculture Guidebook for theCare and Use of Laboratory Animals, Taiwan

Note that full information on the approval of the study protocol must also be provided in the manuscript.
